# Video Q&A: what is ASIA? An interview with Yehuda Shoenfeld

**DOI:** 10.1186/1741-7015-11-118

**Published:** 2013-05-01

**Authors:** Yehuda Shoenfeld

**Affiliations:** 1 Zabludowicz Center for Autoimmune Diseases, Sheba Medical Center, Tel-Aviv University, Tel-Hashomer 52621, Israel

## Abstract

In this Q&A, we talk to Professor Yehuda Shoenfeld about Autoimmune Syndrome Induced by Adjuvants (ASIA) and discuss his recommendations regarding further research in the field.

## Professor Yehuda Shoenfeld talks about ASIA (‘autoimmune syndrome induced by adjuvants’)

## Introduction

Professor Yehuda Shoenfeld is the founder and head of the Zabludowicz Center for Autoimmune Diseases at the Sheba Medical Center, which is affiliated to the Sackler Faculty of Medicine at Tel-Aviv University, Israel. He is also the Incumbent of the Laura Schwarz-Kipp Chair for Research of Autoimmune Diseases at Tel-Aviv University. His clinical and scientific works focus on autoimmune and rheumatic diseases, and he has been the recipient of multiple awards, including a Life Contribution Prize in Internal Medicine in Israel, 2012.

In recent years, Professor Shoenfeld noted that four conditions: siliconosis, Gulf War syndrome (GWS), macrophagicmyofasciitis syndrome (MMF) and post-vaccination phenomena were linked with previous exposure to an adjuvant, and that the patients also presented with similar clinical symptoms. In 2011, this led Professor Shoenfeld to suggest these comparable conditions should be grouped under a common syndrome entitled ‘ASIA’, for ‘Autoimmune (Autoinflammatory) Syndrome Induced by Adjuvants’.

In this Q&A we talk to Professor Shoenfeld about ASIA, and discuss his recommendations regarding further research in the field.

### What is ASIA?

ASIA is a new syndrome, which refers to autoimmune syndromes induced by adjuvants. It includes several conditions that are not fully characterized as autoimmune diseases like systemic lupus, rheumatoid arthritis or scleroderma, but that are induced by chronic stimulation of the immune system by substances which may react as adjuvants. This chronic stimulation leads to the emergence of these new signs and symptoms, which include fatigue, arthritis, myalgia, and neurological manifestations.

### Which adjuvants commonly used in medical practice have been implicated in ASIA?

The idea of ASIA as a new syndrome developed after some studies on Gulf War syndrome reported that soldiers who had not been deployed to the Gulf area were suffering from symptoms such as severe fatigue, cognitive impairment, myalgias and arthralgias. This raised the question of whether it was the vaccines administered to the soldiers that induced these syndromes. The most common adjuvants are silicone implants and aluminum in vaccines.

### Are any other adjuvants associated with ASIA?

There are some specific adjuvants which have been shown to induce ASIA; for instance, aluminium. Aluminium is the oldest, the cheapest and the most efficient adjuvant so far, which is why it is still commonly used in the development of vaccines.

In 2001, Romain Gherardi and colleagues reported that patients diagnosed with macrophagic myofasciitis, or MMF (a rare muscle disease characterized by specific myopathological alterations, first described by the Groupe d’Etudes et Recherche sur les Maladies Musculaires Acquises et Dysimmunitaires (GERMMAD)), had previously been vaccinated with hepatitis vaccines containing aluminium hydroxide. These patients went on to develop severe myalgia with neurological manifestations, cognitive impairment, dizziness, inability to concentrate and poor sleep. Following many studies, Romain Gherardi and colleagues were able to demonstrate that the aluminium is deposited in the muscle, and then via macrophages travels from the muscles to different organs and penetrates the blood-brain barrier. On this basis, MMF is part of the ASIA syndrome.

Another condition termed the ‘sick building syndrome’ (SBS) leads to similar clinical symptoms as the Gulf War syndrome, and manifests in people living in a specific room or building. However, once they move to another room or to another building, they completely recover. It is believed that, in that room, there is some substance that reacts or behaves like the adjuvant. I have already mentioned that aluminium is used as an adjuvant in vaccines, but as one of the most common materials in the world its uses are even more widespread, as much of the equipment we use in our daily lives is made from aluminium.

### As the use of vaccines, silicone implants, etc., is widespread, what does this mean in terms of public health?

First of all, vaccines are *very* widespread, and I would like to clarify that I am definitely not against vaccines! Vaccines are the best medical development that humankind has had in the last 300 years, and have helped to bring about almost complete eradication of some viral diseases. However, it should be considered that when you give millions of people an active substance, and vaccines are active substances, then some may suffer from adverse events. After all, vaccines contain viral or synthetic particles emulsified in adjuvant, which is supposed to enhance the immune reaction.

So, we have to identify the people who are at risk of suffering from side effects due to the chronic stimulation of their immune system. First of all we have to diagnose them, to treat them—and some of them should be compensated, because the vaccines are quite often imposed on them either by the state, the government, or by the employer.

With regard to silicone implants, which is a very common cosmetic operation, there have been claims that the silicone is completely inert, that it doesn’t leak and travel through the body, that it doesn’t induce granulomas, etc., but this is misleading. There have been recent cases of ruptured silicone implants, but even with unruptured implants, there are nanoparticles of silicone that can travel through the body. Therefore silicone can be found in different parts, such as the hands, the chest and the groin. Although silicone implants are quite common, luckily enough the syndrome itself is rare.

Similarly, autoimmune diseases which are definitely induced by infections are not as common as the infections themselves. This is because a specific interaction between the infective agent and the genetic components is linked to incidence of autoimmune diseases.

With regard to the ASIA syndrome, prevalence is higher in subjects that carry the gene HLA-DRB1. It should be noted that this is the same HLA (human leukocyte antigen) which was found to be present in those who had developed an autoimmune disease following vaccine administration. So, maybe in the future with further advances in personalized medicine, we will be able to screen those at risk based on their genetic composition, and therefore avoid onset of autoimmune diseases by avoiding administration of vaccines containing adjuvants that are known to be associated with the ASIA syndrome. In test subjects the type of adjuvant can be replaced with one that might not be associated with the ASIA syndrome. I would like to emphasize that currently there are novel adjuvants in development which have to be tested for efficacy, but we hope these might have fewer side effects than aluminium and other established adjuvants.

### What are the current criteria used for diagnosis of ASIA?

We have published the criteria and classified it as we do usually with different autoimmune diseases, namely into major criteria and minor criteria. Major criteria include clinical manifestations such as severe fatigue, poor sleep, myalgia and arthralgia; the minor criteria include the presence of various autoantibodies and specific HLA (e.g., DRB1). However, as I mentioned before, over the years many of these patients may go on to develop a more well-defined autoimmune disease. For instance, if they develop scleroderma or systemic sclerosis, they will suffer from tight skin, complications of the lungs, kidneys, and so forth.

### Have the mechanisms via which adjuvants may cause these effects been established?

In part, the mechanism involves the chronic stimulation of the immune system, which may then lead to the release of inflammatory cytokines including interferon γ, interferon α, interleukin (IL)-1, IL-6, tumor necrosis factor (TNF)α and so forth. So in part, this syndrome can be induced by this cascade of cytokines released in response to the chronic stimulation.

This chronic stimulation may also involve the opening of the blood-brain barrier, and therefore penetration of different substances into the brain. For example, one of the mechanisms which has been well defined is that aluminium deposits in the body following vaccine injection, or following exposure to other sources of aluminium, and these particles can cross the blood-brain barrier via macrophages and deposit in the brain.

In the past, we physicians witnessed this aluminium ‘toxicity’ of the brain in cases where the patient went through dialysis. The dialysate fluid contained aluminium, and actually diffused into the brain through the blood-brain barrier leading to aluminium intoxication. In some cases, the patient presented with symptoms consistent with the ASIA syndrome.

In addition, chronic stimulation also induces different autoantibodies. Although the autoantibodies do not necessarily indicate a specific autoimmune disease, it might be a combination of anti-DNA antibody which is more classic for systemic lupus erythematosus (SLE), but also anti-mitochondrial antibodies which may indicate primary biliary cirrhosis. As I mentioned earlier, the undefined connective tissue diseases (UCTD) over the years may develop to a specific autoimmune disease. Therefore we also believe that most cases of UCTD are actually part of ASIA syndrome.

### Is there any evidence to suggest that environmental or genetic factors may lead to higher risk of developing ASIA?

The actual prevalence of environmental factors, in addition to silicone implants and the adjuvants in the vaccine, is not yet known. But based on my experience and from reading the literature, I can assume that aluminium and other different materials used in daily life may be associated. For instance, in the 1980s a significant increase in lupus cases was observed in an ex-industrial town in the United States: East Ferry Street in Buffalo, New York. Following investigations it was found that the area was heavily contaminated with toxic materials (including lead, polychlorinated biphenyls, trichloroethylene and volatile organic compounds).

So, looking at this from a wider context I would say that it can be considered that toxic materials which can act like the adjuvants may underlie what we call ‘idiopathic’ autoimmune diseases.

### What is known about the prevalence of ASIA, particularly in terms of geographical distribution?

There is no knowledge about geographical distribution. We do know that many of the autoimmune diseases are more prevalent in populations that live further away from the equator. It is believed that limited exposure to sun, and therefore the lack of production of vitamin D, may be associated with ASIA. We know that vitamin D is associated with many autoimmune diseases. For instance, in one study we analyzed more than 40 different autoimmune diseases, and found the patients had significantly lower levels of vitamin D in comparison with the healthy population within the same geographical area.

Studies have emerged from the Philippines, Mexico, from all over the world, but these are just small series of cases. There are no large epidemiological studies so far that have been able to analyze the geographical distribution. However, I believe that eventually we will find a correlation between the ASIA syndrome and geographical distribution.

One study from Finland reported a large increase in cases of narcolepsy, which is now recognized as an autoimmune condition. The researchers correlated this increase of narcolepsy incidences with the vaccines which were administered during the H1N1 (swine flu) epidemic in that region. I would like to emphasize that this disease is recognized in Finland and is strictly associated with the (HLA) DQB1*0602 genotype. When the vaccine was delivered during the H1N1 epidemic, there was a 13-fold increase of narcolepsy in this geographical area. So the geographic distribution in this case was not connected to the substance, but rather to the genotype of the people who live in the area, which made them more susceptible to developing narcolepsy.

### What do you think are the future directions of research into this field?

Efforts should be made to understand the mechanisms behind ASIA and to develop better adjuvants, especially in vaccines. After all, numerous people are vaccinated regularly, and we should minimize any potential side effects.

We should learn from ASIA to better understand the etiology of other autoimmune diseases that are currently regarded as ‘idiopathic’ (which means that we are idiots as we don’t know the pathology and etiology!). I would like that in the future, I will not see the sentence ‘autoimmune diseases have an unknown etiology’, because we are coming closer to better understanding this.

I am still concerned about silicone implants; whether to recommend these should be explanted or not, as there is no guarantee of full recovery from the ASIA syndrome if the silicone implants are explanted. If not, then the patient is left without the implants and continue to suffer from the ASIA syndrome. Having said that however, there are a few cases where explanting led to a complete recovery of the patient from the ASIA syndrome. This requires further investigation.

A lot of research is going on all over the world, and I hope that in the future we’ll be able to better identify those who are at risk of developing this syndrome, and to avoid it before they do.

### What is your advice to clinicians for the management and treatment of patients with autoimmune disorders? Should they be routinely screened for ASIA?

As there are no markers for ASIA, we cannot screen for this. My advice to the clinician is to pay more attention to the history of the patient, specifically regarding their vaccine history. In the future, I would like for clinicians to be able to diagnose the patient early on, and in addition may help to compensate them if appropriate; after all, these patients suffer. They were completely healthy until they were vaccinated and then suddenly developed the disease.

Better understanding of the symptoms and development of serological markers may help to identify the risk factors such as HLA and familial autoimmune background at a very early stage. The therapy for ASIA should be the same therapy as for autoimmune diseases—and until we can understand it better, maybe clinicians should consider switching to other biological drugs that have not been associated with ASIA, rather than continue to use the drugs that have been.

### Where can I find out more?

See references [1-28].

## Pre-publication history

The pre-publication history for this paper can be accessed here:

http://www.biomedcentral.com/1741-7015/11/118/prepub

## Supplementary Material

Professor Yehuda Shoenfeld talks about ASIA (‘autoimmune syndrome induced by adjuvants’) Click here for file
